# Ablation for Single Pulmonary Nodules, Primary or Metastatic. Εndobronchial Ablation Systems or Percutaneous

**DOI:** 10.7150/jca.90494

**Published:** 2024-01-01

**Authors:** Paul Zarogoulidis, Vasilis Papadopoulos, Eleni-Isidora Perdikouri, Anastasios Vagionas, Dimitris Matthaios, Aris Ioannidis, Wolfgang Hohemforst-Schmidt, Haidong Huang, Chong Bai, Oikonomou Panagoula, Christina Nikolaou, Charalampos Charalampidis, Christoforos Kosmidis, Konstantinos Sapalidis, Nikolaos Machairiotis, Athanasia Pataka

**Affiliations:** 1 Pulmonary Department, General Clinic Euromedica, Thessaloniki, Greece.; 2 Oncology Department, University General Hospital of Larissa, Greece.; 3 Oncology Department, General Hospital of Volos, Greece.; 4 Oncology Department, General Hospital of Kavala, Greece.; 5 Oncology Department, General Hospital of Rhodes, Greece.; 6 Surgery Department, Genisis Private Clinic, Thessaloniki, Greece.; 7 Med IV IP, Lung Centre Bamberg Clinics aff. Univ.- of Erlangen, Buger Str. 96049 Bamberg Germany.; 8 Department of Respiratory and Critical Care Medicine, Changhai Hospital, Navy Military Medical University, Shanghai, 200433, China.; 9 Surgery Department, Democritus University of Thrace, Alexandroupolis, Greece.; 10 Department of Pathology, University of Cyprus, Cyprus.; 11 3rd University Surgery Department, ``AHEPA`` University Hospital, Thessaloniki, Greece.; 12 Third Department of Obstetrics and Gynecology, University General Hospital “ATTIKON”, Medical School of the National and Kapodistrian University of Athens, Athens, Greece.; 13 Pulmonary Department, G. Papanikolaou General Hospital, Aristotle University of Thessaloniki, Greece.

**Keywords:** Lung Cancer, NSCLC, ablation, radial-ebus, CBCT, Monarch, Archimedes, Covidien, Medronic, biopsy, ROSE, microwave, radiofrequency, thermopshere, cryo, Cone beam computed tomography

## Abstract

Single pulmonary nodules are a difficult to diagnose imagining artifact. Currently novel diagnostic tools such as Radial-EBUS with or not C-ARM flouroscopy, electromagnetic navigation systems, robotic bronchoscopy and cone beam-compuer tomography (CBCT) can assist in the optimal guidance of biopsy equipment. After diagnosis of lung cancer or metastatic disease as pulmonary nodule, then surgery or ablation methods as local treatment can be applied. The percutaneous ablation systems under computed tomography guidance with radiofrequency, microwave, cryo and thermosphere have been used for several years. In the past 10 years extensive research has been made for endobronchial ablation systems and methods. We will present and comment on the two different ablation methods and present up to date data.

## Introduction

Lung cancer is usually diagnosed at advanced inoperable stage. A very important reason is the lack of early disease symptoms and lack of blood tests as we have in other cancer types such as breast cancer, prostate cancer and gastrointestinal cancer. Very few patients will present hemoptysis in early stage disease due to endobronchial disease and will search for medical evaluation. Moreover; most patients since they are smokers will attribute their progressive dyspnea to their smoking habit and chronic obstructive disease (COPD) and will seek for medical attention when their clinical status is very severe. There are also cases where pulmonary nodules are observed during re-staging for other cancers types such as; breast cancer, gastrointestinal or prostate cancer. In the past 10 years there was a bloom regarding navigation technologies for single pulmonary nodules. Radial-endobronchial ultrasound has been used with or without C-ARM fluoroscopy for guidance assistance 1. Electromagnetic technologies with Medronic and virtual bronchoscopy navigation with Archimedes are also in the market in the past 10 years with minor differences between them 2-4. C-Arm fluoroscopy can be also used when performing electromagnetic guidance. CBCT can be used with another realtime method 5, 6. Rapid on site evaluation (ROSE) is a method where cytology samples obtained from the biopsy procedure can identify whether there is cancer in the nodule and whether the sample is enough for further investigation for immunohistochemistry and gene expression 7. Robotic bronchoscopy has been introduced in 2018 with Ion and Monarch platforms 8, 9. Ablation as a local treatment has been introduced in the past 20 years and different generators and methods are used such as the radiofrequency, microwave, thermosphere and cryo. Percutaneous and surgical probes are also available based on the location of the lesion 10-13. The usual side effects are pneumothorax, hemoptysis or even hempthorax. A recent meta-analysis presented data where cryo ablation is superior to radiofrequency ablation, but not microwave ablation 12. In the past 10 years new endobronchial ablation systems have been introduced in the market such as the Bronchus which is a radiofrequency catheter guided by Archimedes electromagnetic navigation systems and NEUWAVE^TM^ FLEX Microwave Ablation System guided by the MONARCH® Platform by Ethicon. There are certain advantages for each methodology that will be discussed in the section that follow.

## Materials and Methods

We made a thorough search of the literature on pub med for new publications using strictly the following key words: radiofrequency ablation systems for lung cancer, microwave ablation systems for lung cancer, cryo ablation systems for lung cancer, thermosphere ablation systems for lung cancer, endobronchial ablation systems for lung cancer. We identified 35 publications, including case reports and written in all languages and used the data from 28. We focused our manuscript strictly on these data presenting up to date data.

### Percutaneous ablation systems

Radiofrequency ablation with percutaneous probes for lung cancer has been well established since 2000 and there are numerous studies with the effectiveness of this technique 14. The next equipment to be validated for lung cancer was microwave generators 11. There are differences between the radiofrequency and the microwave effect, and methodology. Microwave technology was observed to be more efficient than radiofrequency 15. Currently the thermal effect of microwave application has been enhanced by adding transbronchial thermal gel 16. Moreover; thermosphere technology combined with microwave ablation has increased the efficiency of the method 13. Cryo ablation is the latest technology on the field of local percutaneous treatment under computed tomography guidance 17. The effect of the cryo ablation is superior to radiofrequency, however; it is not superior to microwave ablation 12.

### Navigation for endobronchial ablation systems

The main issue with single pulmonary nodules is the diagnosis. There are 6 distinct reasons to go early for small malignant nodules: a) The lower the T the better the survival (weak relation) acc. to TNM V8 and the lower the probability of N1 and yet the probability of local relapse, b) The smaller the nodule even in the same T-descriptor (e.g. Stage I) the better the long term survival, c) The smaller the nodule the lesser the intratumoral heterogeneity and the mutational probability which is an independent risk factor of relapse, d) No / less reduction of postinterventional lung function loss after minimal invasive approach should pave the way to more options in possible return of cancer and e) The smaller the nodule the better is add-on surgery / SBRT / transthoracic ablation (The KISS) or drug treatment like EPR, ITC and TBNI if not possible ot treat endobronchially completely.

**Figure 2:**
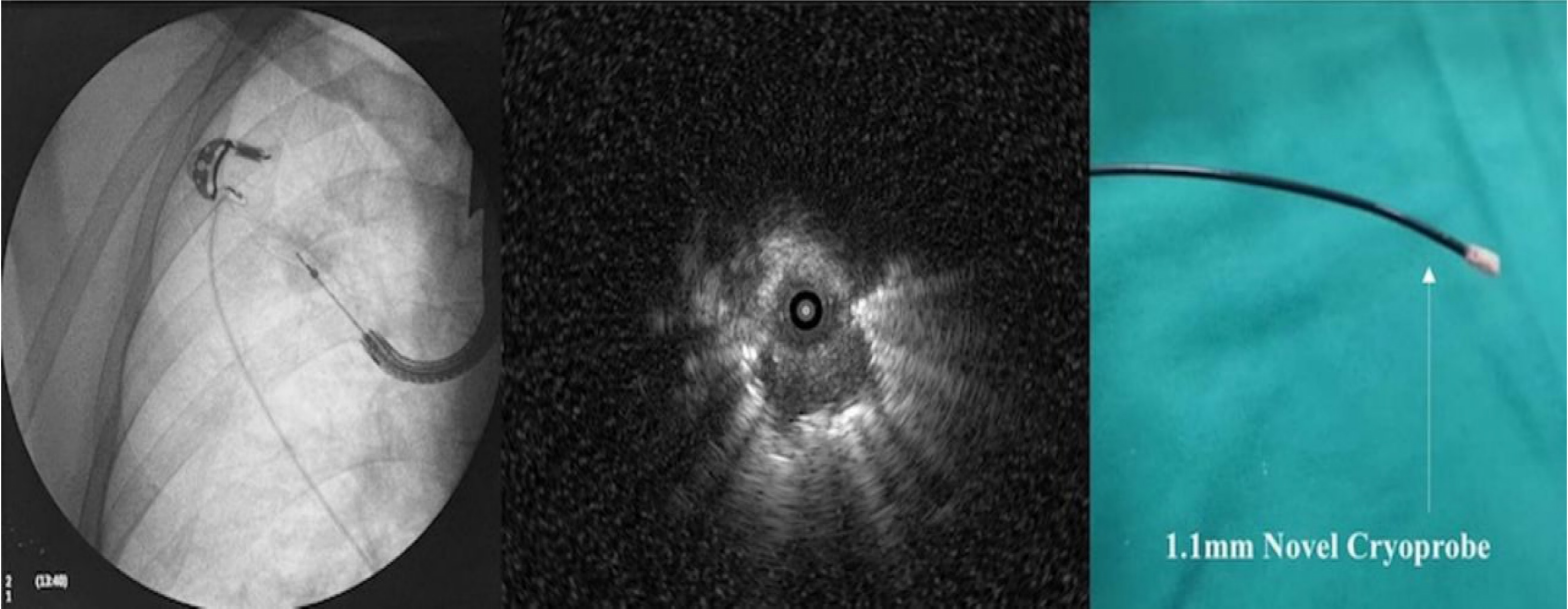
Left; Flouroscopic guidance of cryo probe 1.1mm, Middle; radial ebus image from the pulmonary nodule, right; cryoprobe 1.1mm.

**Figure 3:**
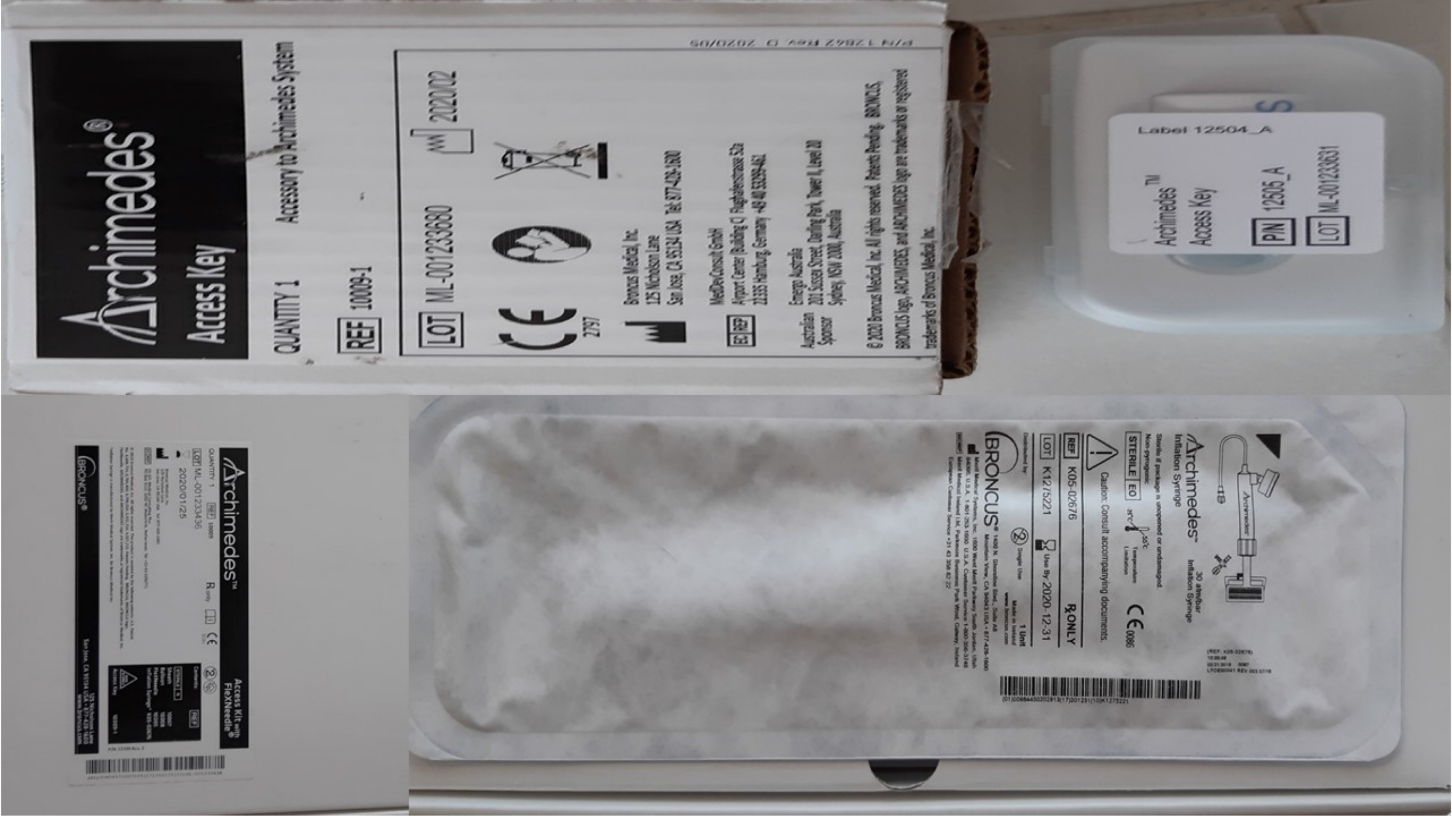
Archemedes, Bronchus Diagnostic catheter (photo provided by Paul Zarogoulidis).

**Figure 4:**
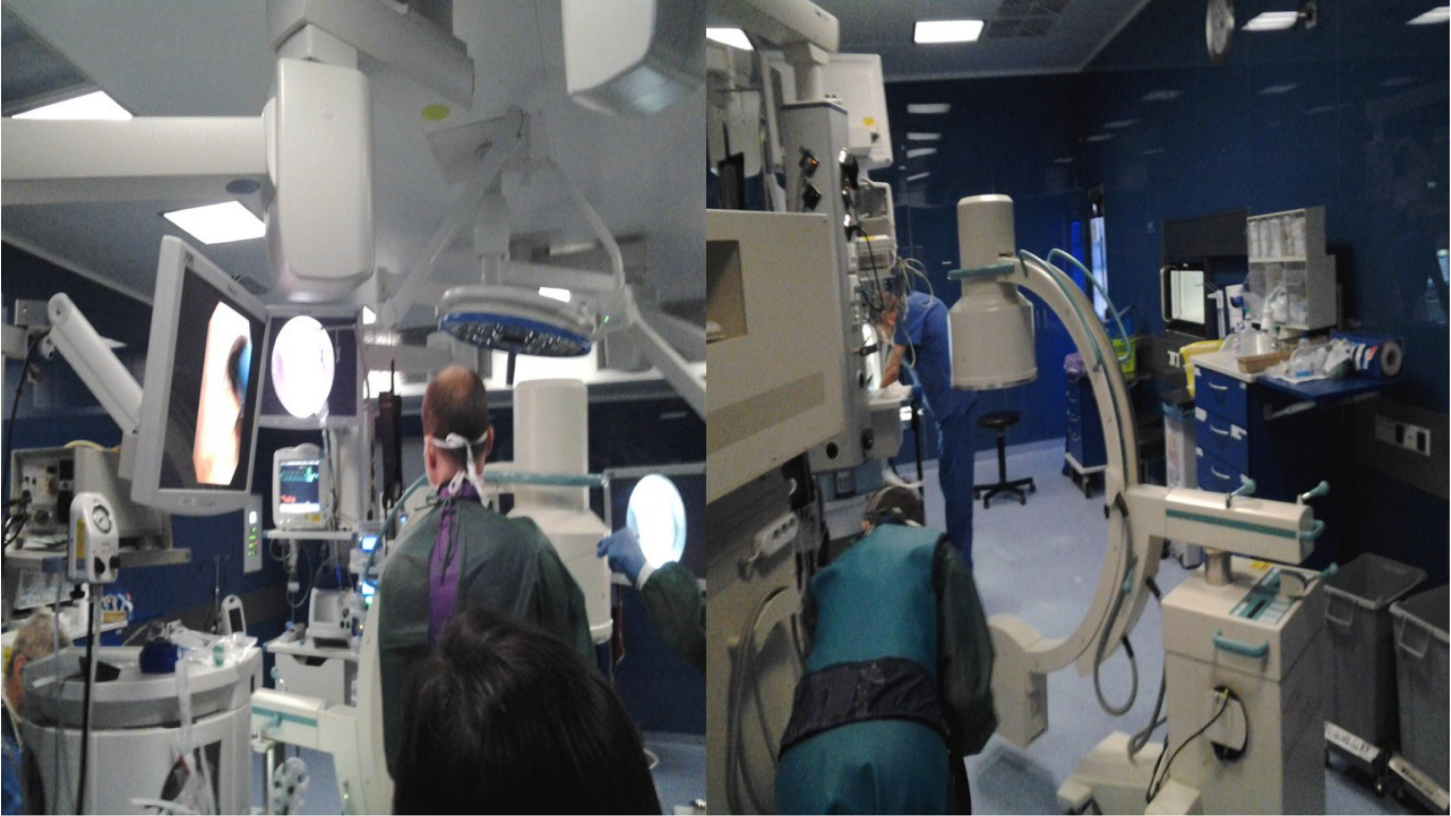
C-Arm fluoroscopic guidance for radial ebus.

**Figure 5:**
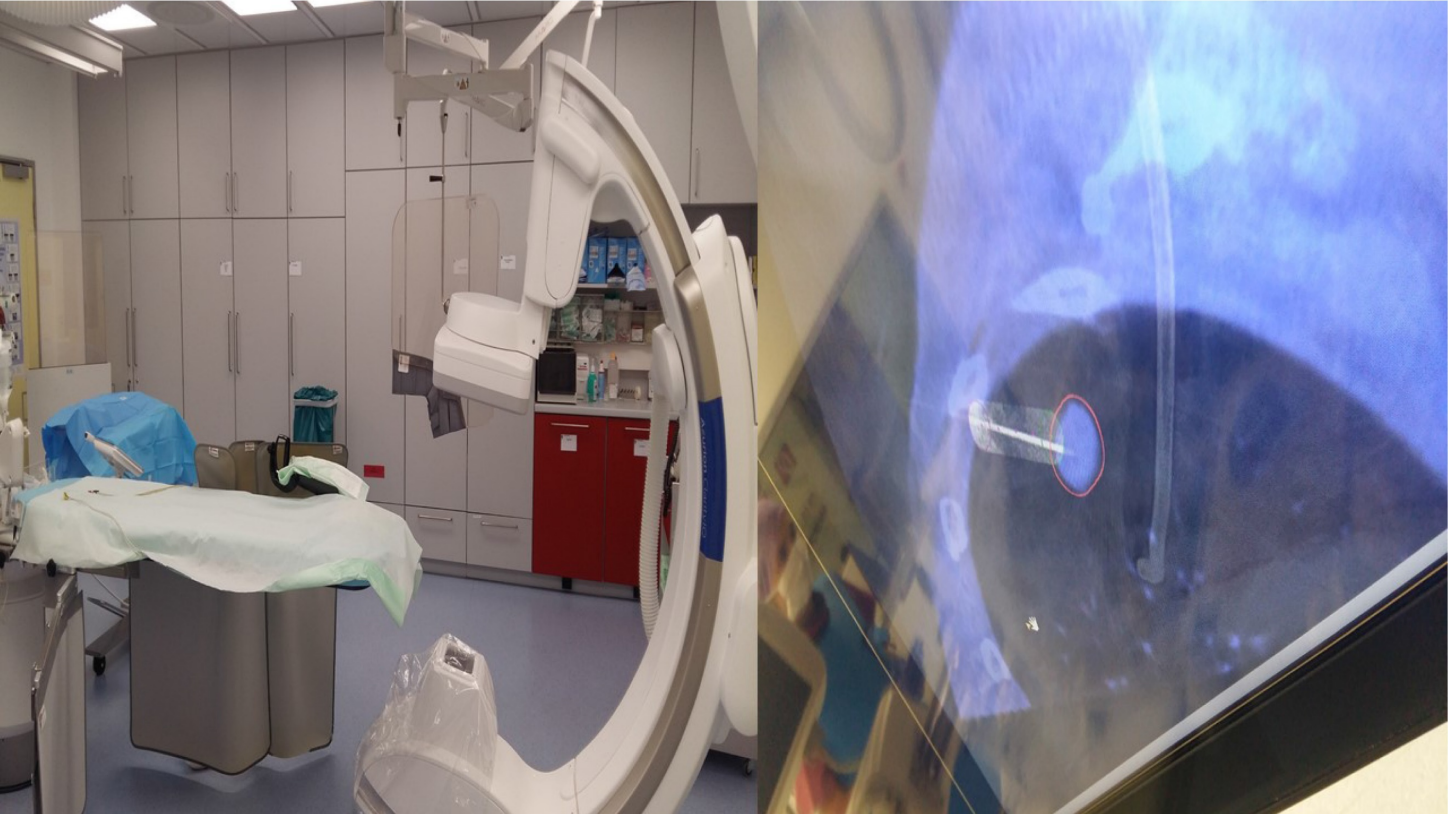
Cios Spin Siemens.

We currently use radial-EBUS with or without C-ARM fluoroscopy or in combination with other navigation systems 1. The main biopsy tools are cytology brush, mini forceps, fine needle aspiration (22G) and thin cryo probes (Figure 2). We have even the capability of making tunnels through the lung parenchyma with needles and balloon dilation and obtain sample 18, 19. This method is safe and effective when navigation systems are used such as the Archimedes Virtual Bronchoscopy Navigation (VBN) (Figure 3), Illumisite^TM^ platform from Medronic 20. Another novel navigation technique is the robotic assisted navigation with the Monarch^®^ Platform (Auris Health, Inc., Redwood City, CA) 8. Other combination techniques and equipment is currently available that can be used with radial-ebus such as the Cios Spin^®^ by Siemens Healthineers^21^, O-arm^TM^ O2 imaging system by Medtronic and Super Dimension^TM^ (Covidien, Plymouth, MN, USA) 22, 23. Cone-beam CT apparatus (ARTIS zeego; Siemens Healthcare GmbH, Erlangen, Germany) is another equipment-technique that can be also used 24 (Figure 4,5). Several studies have been made with all these equipment, however; higher diagnostic efficiency was observed for those studies with nodules ≥30mm.

**Figure 6:**
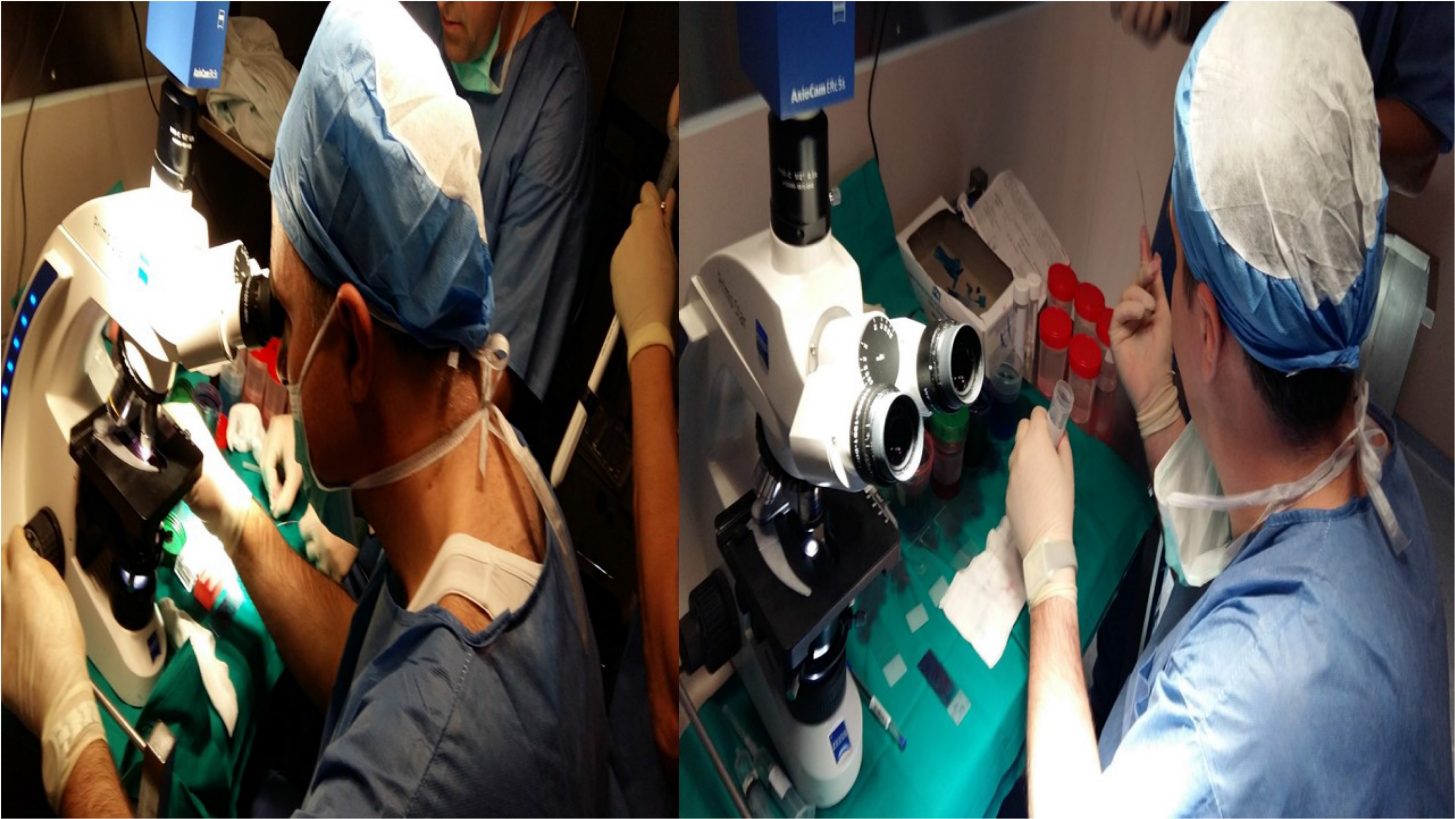
Rapid on site evaluation from convex endobronchial ultrasound with 22G fine needle aspiration biopsy (Mediglobe needle).

**Figure 7:**
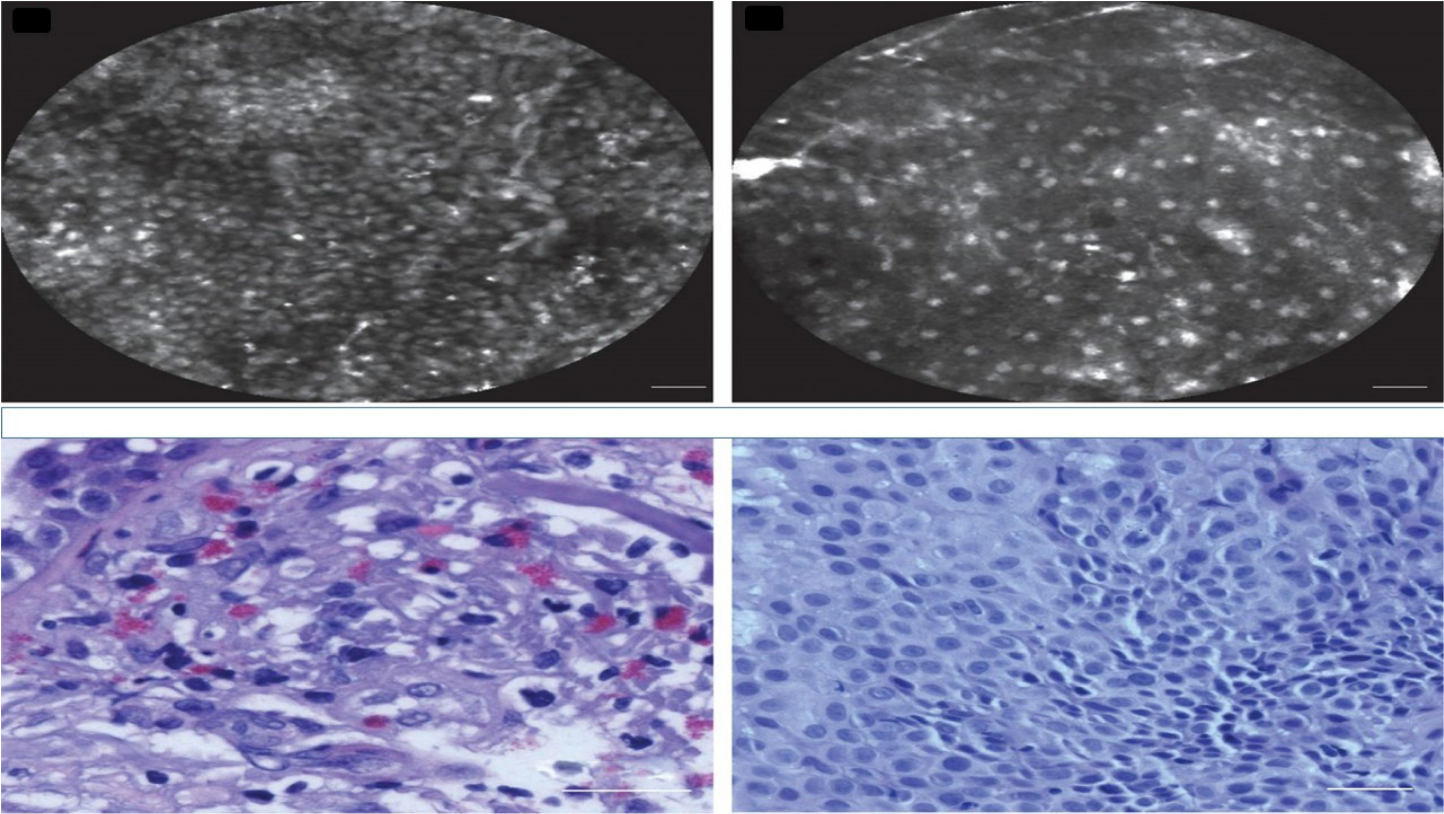
Confocal laser endomicroscopy, upper row confocal image, lower row cytology image. Left; squamous cell carcinoma, Right; adenocarcinoma. Cellvizio Mauna kea technologies.

A recent paper has shown in a meta-analysis that CBCT is as stand-alone as well in combination with other technologies superior to all other technologies stand-alone or in combination. It is as well cost-effective in regards to QUALYS in the Dutch Healthcare System versus transthoracical approach 25, 26. Rapid on-site evaluation was used to increase the diagnostic yield in some studies 27, or confocal laser endomicroscopy 28 (Figure 6,7).

### Endobronchial ablation systems

Radiofrequency ablation systems are already in the market by Brochus and there are several studies 29, 30. A microwave catheter (Emprint^TM^ ablation catheter with the ThermosphereTM technology, Covidien, Plymouth, MN, USA) is already on the market. Flexible water-cooled MWA antenna (Vison-China Medical Devices R&D Center) connected to a microwave platform (Surblate, Vison) and an MWA device for ablation by Nanjing Nisionmedic (Nanjing, China) 31-34. The first-in-the-world ENB microwave ablation using the Illumisite^TM^ fluoroscopic navigation platform was successfully performed in mid-2022 35.

### New methods for ablation

Cryo ablation probes are available for percutaneous application but, not for transbronchial application, although one could use in certain cases the available probes from ERBE II system 36. Robotic assisted guided cryobiopsy has been presented in a previous study and it could be the platform for future application of a cryoprobe system 37. There have been pilot studies evaluating *in vitro* a novel transbronchial cryo probe 38, 39. However; we still need human studies. Bronchoscopic thermal vapor ablation (BTVA) by Bronchus is available since 2021 for emphysema treatment, recently a study demonstrated efficiency when this technique is used as an ablation tool 40, 41. Further studies are required to explore and improve on it before it can be reliably used for cancer treatment. Until now microwave ablation systems are available for percutaneous usage, however; there are systems being tested for transbronchial treatment in animal models 42. Pulsed electric field (PEF) is a non-thermal ablative modality that uses a short-living strong electrical field created around a catheter to create microscopic pores in cell membranes (electroporation). Finally, a device with radiofrequency ablation catheter has been investigated which is compatible with endobronchial ultrasound 43.

### The use of rapid on-site evaluation (ROSE)

Due to the increasing rate of computed tomography scans based on the new screening guidelines for lung cancer, more and more patients are diagnosed with pulmonary nodules. It is absolutely necessary to identify whether we have or not malignancy. We can use positron emission tomography scan (PET-CT) as an initial examination, however, there is still a diagnostic issue correlated with the size of the nodules. In the case of nodules ≤1.2cm and with a low metabolic rate ki-67≤10% the technique cannot provide accurate data even with a second delayed reexamination after 30minutes 44. Therefore biopsy is absolutely necessary in the case of a PET-CT examination with low metabolic rate ≤3SUV. Novel diagnostic techniques can provide adequate navigation and diagnostic yield for pulmonary nodules ≤1.2cm. However; the sample will mostly be cytologic, since a cytology brush or needle will be used. We can use of course where possible biopsy forceps or small cryo-probes 1.1mm in order to obtain tissue. Again the sample will be small is quantity, but not in quality. We can verify the malignancy with rapid on-site evaluation and the quality of the sample (whether we can perform additional immunohistochemical examinations). After biopsy we need approximately 2-5 minutes to prepare the sample for evaluation in the microscope. We perform evaluation of at least two samples from the same site 45.

## Discussion

Currently we propose a screening methodology to smokers, ex-smokers or people of high risk for lung cancer 46. Therefore, we have more patients diagnosed with pulmonary nodules. In the past ten years we have had both a bloom in novel diagnostic techniques for pulmonary nodules and treatment for advanced stage lung cancer. The radial-ebus along with fluoroscopic techniques such as C-ARM, DYNA-CT, O-ARM and Cios-Spin have achieved increased navigation and diagnostic yield. Electromagnetic platforms such the Archemedes^®^, Illumisite^TM^, and *Veran's SPiN Thoracic Navigation System* platform have increased even more our navigation and diagnostic yield in pulmonary nodules ≤30mm. We have now the capability to identify the location of vessels during our diagnostic procedure. Robotic assisted bronchoscopy has been also introduced in 2018 with Monarch and Ion platforms, each one with characteristics. Both systems have equal navigation and diagnostic results. Along to all these navigation systems we can use additional methods of rapid on-site evaluation to evaluate our samples and complete a diagnostic sequence earlier. The rapid on-site evaluation has been established more than 5 years and requires a learning curve from the bronchoscopy operator, or a cytologist can be used at the site of diagnosis. Confocal laser endomicroscopy can be also used as a rapid on-site tool, again a learning curve is required, or a cytologist on site. Since we have the ability to diagnose early stage disease, lung cancer or metastatic disease, we can use advanced systems for minimal local therapy. Local treatment for malignant pulmonary nodules either primary lung cancer or metastatic is efficient either percutaneously under computed tomography guidance with radiofrequency, microwave or cryo-ablation systems. Currently there are many studies presenting the efficiency and adverse effects of these systems and methods. The indications are also very specific and well known. There are several other treatment modalities such as stereotactic body radiotherapy or radiotherapy with or without systematic administration of drugs 47 48. These treatments have a different application and results from local ablation, it remains for the treating physician to choose the best method of local disease control best on the clinical features of the patient. Radiotherapies are known to have adverse effects such pneumonitis or esophagitis. Moreover; application of this treatment modality is excluded when vessels are near the lesion. Local percutaneous systems have as major adverse effect pneumothorax and hemothorax. The pneumothorax if less than 1lt can be treated on site with a pneumocatheter, if more than 1lt then with a bullau insertion, however; several days of hospitilisation will be necessary. Indeed, due to severe emphysema, the application of percutaneous ablation might not be possible. Transbronchial ablation is an option now more than ever since we have efficient navigation. We have tools to minimize hemorrhage by spraying polymer dust to stop bleeding or in the case of fistulas we can use stents or even emphysema valves to block a sublobar segment. A balloon dilation system can also be applied to block the hemorrhage. The balloon blockers can stay inside a patient for up to 7 days if necessary. Moreover; we can eliminate most malignant pulmonary nodules as a `One Stop Shop`, since we have rapid on-site diagnosis available. Positron emission computed tomography (PET-CT) can provide staging for patients and possible biopsy sites. However; again for small pulmonary nodules ≤1.2cm again for those lesions with low metabolic rate ki-67 ≤10% we need again biopsy.

In the case where a pulmonary nodule is more than ≥3cm then adjuvant systematic therapy should be used. In specific for these patients with non-small cell lung cancer (NSCLC) we have to identify the subtype adenocarcinoma, squamous cell carcinoma or where this is not possible non-other specific (NOS). First line targeted treatment with tyrosine kinase inhibitors (TKIs) are based on the expression of epidermal growth factor receptor (EGFR), anaplastic lymphoma kinase (ALK) and proto-oncogene 1 (ROS-1) 49, 50. Regarding EGFR positive patients, mutations were observed such as T790M and therefore second line tyrosine kinase inhibitors (osimertinib) were produced, which are now administered as first line 51. However; again resistance to osimertinib was observed, and in this case another inhibitor (of the MET pathway) capmatinib was observed to overcome this resistance by decreasing the generation of cancer-associated fibroblasts. Capmatinib suppresses the MET/akt/snail signaling pathway 52. Moreover; v-Raf-murine-sarcoma-viral-oncogene-homolog B (BRAF V600E), neurotrophic-tyrosine-receptor-kinase (NTRK 1/2/3) gene-fusion, mesenchymal-epithelial-transition exon14 skipping (MET) and re-arranged-during-transfection (RET) rearrangement are also novel gene expressions targeted 53. Regarding immunotherapy we investigate the programmed death-ligand 1 (PD-L1) expression in order to administer immunotherapy alone (PD-L1≥50%) or in combination with chemotherapy (PD-L1≤49%) 54. In the recent years based on preclinical and clinical trials other mutations such Kirsten rat sarcoma (KRAS), amplification of human epidermal growth factor receptor-2 (HER2), and other genotypes of the driver genes, have been thought highly targetable 55. We use next-generation sequencing (NGS) panel to identify gene expressions 49. Tissue biopsies remain the gold standard for gene expression identification, however; in the case where this is not possible, we can use liquid biopsies which can detect circulating tumor DNA (ctDNA) 56.

**Figure 8:**
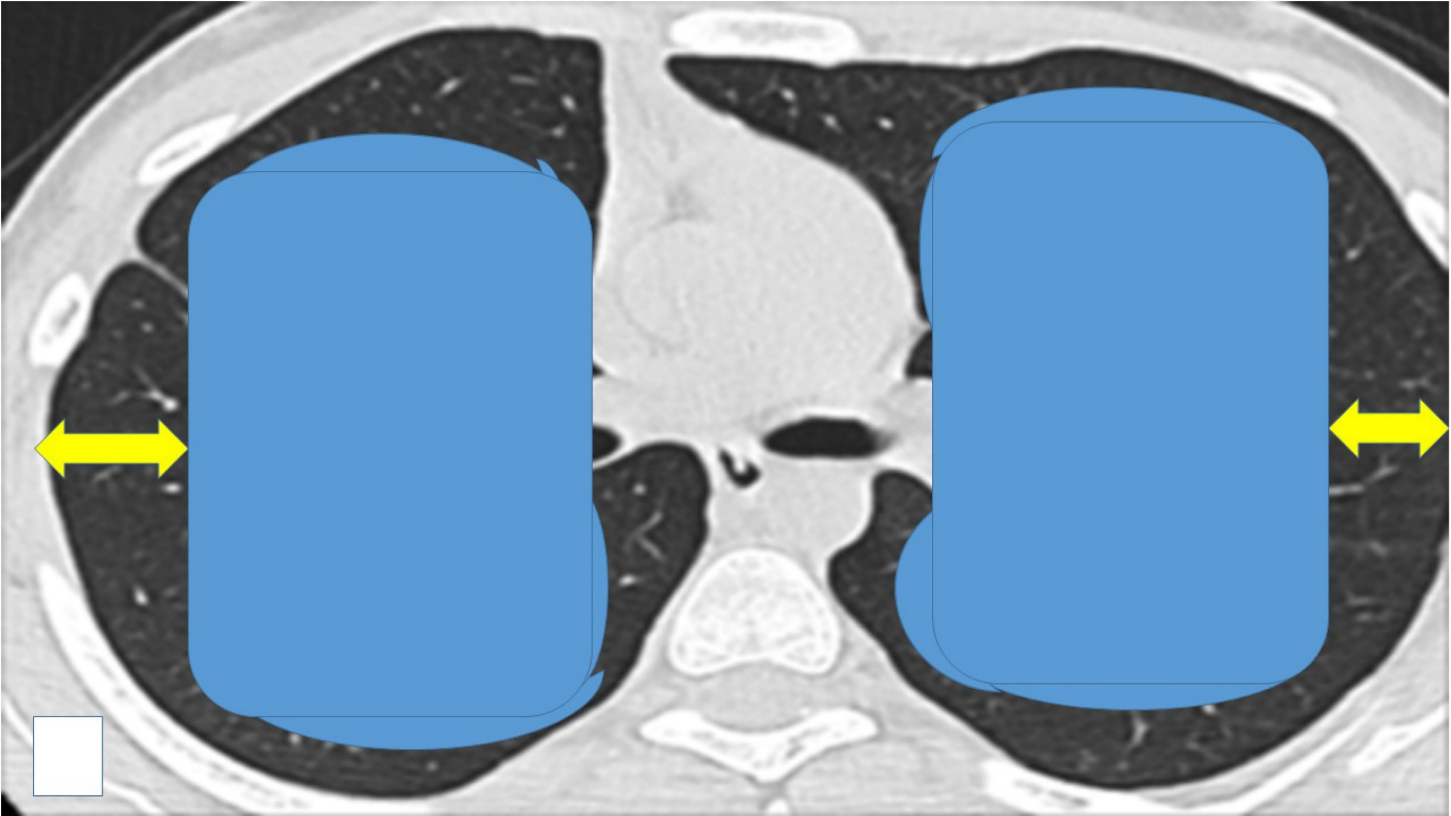
We propose that the endobronchial-transbronchial ablation is mainly used inside the blue areas, while the percutaneous in the black areas (yellow arrows). In order to choose the appropriate ablation system the following information should be considered: a) center experience, b) cost-effectiveness, c) site of lesion (surrounding vessels or bronchus), d) size of lesion, e) available navigation equipment, f) ablation effectiveness for lung lesions and g) number of lesions. Also, an additional insight should be the origin of the lesion, kidney cancer needs more ablation time than squamous cell lung carcinoma. Finally, the center should be able to handle all adverse effects, such hemothorax, pneumothorax, fistulas, respiratory distress and must have intensive care unit.

There have been numerous studies where ablation systems have been used along with the administration of local or systematic drugs 57-59. We already have sufficient data that demonstrate the synergistic effect of ablation systems with the co-administration of drugs and this will be the future direction for research in the field of local treatment 15, 60-62. Moreover; our group has evaluated a radiofrequency ablation catheter for vessels from Covidien in order to treat small malignant pulmonary nodules 63, 64. This catheter with minor adjustments such as the addition of a spick tip can be better inserted in pulmonary lesion. The effect of the transbronchial ablation can be identified with radial ebus before and after the ablation effect and possibly by using an additional software such as elastography. Compared to other forms of thermal energy, microwave ablation is the most promising one based on the currently available early to midterm results. We present in our final figure our proposal for transbronchial ablation candidates versus percutaneous ablation based on the site of the pulmonary nodules (Figure 8).

## Figures and Tables

**Figure 1:**
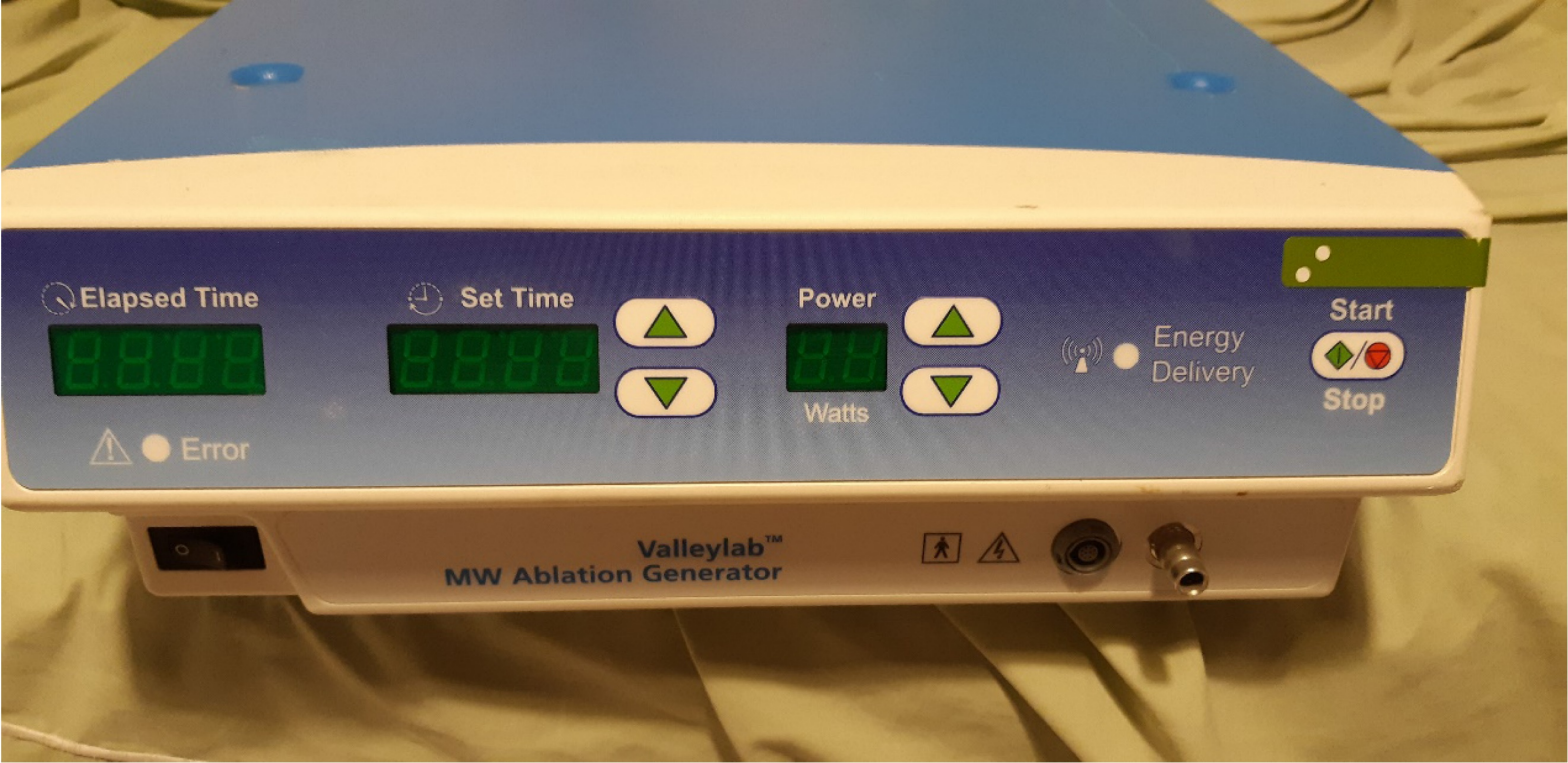
Covidien microwave generator (Photo provided by Paul Zarogoulidis).
